# Breast Angiosarcoma: 15‐Years Experience

**DOI:** 10.1111/ans.70673

**Published:** 2026-04-16

**Authors:** Gaik Si Quah, Christopher Pyke, Christopher Allan

**Affiliations:** ^1^ Mater Brisbane Hospital Brisbane Queensland Australia; ^2^ University of Queensland Brisbane Queensland Australia

## Abstract

**Background:**

Breast angiosarcoma is a rare, aggressive malignancy that can be primary (PAS) or secondary (SAS). This study evaluates the management and outcomes of breast angiosarcoma in a single‐center breast/surgical oncology unit.

**Methods:**

A retrospective review was conducted on patients with histologically confirmed PAS or SAS who underwent surgical resection at Mater Hospital Brisbane between 2009 and 2024. Patient characteristics, clinical features, treatment, complications, and survival outcomes were analyzed.

**Results:**

Fifteen patients were included—4 with PAS and 11 with SAS. SAS patients were significantly older, all had a history of breast cancer treated with adjuvant radiotherapy, and commonly presented with skin discoloration (bruising or violaceous rash). There was a trend toward a shorter duration from presentation to diagnosis and fewer investigations required in PAS patients. Management did not differ between PAS and SAS groups; all patients underwent surgical resection and less than half received adjuvant radiotherapy or chemotherapy. Median follow‐up was 27 months for PAS patients and 12 months for SAS patients. The local recurrence rate in the SAS group was 9.1%, with no local recurrence observed in the PAS group (*p* = 0.53). Mortality rates were 25% in the PAS group and 18.2% in the SAS group (*p* = 0.77).

**Conclusion:**

Breast angiosarcoma has a poor prognosis with high recurrence and mortality. Diagnosis is difficult due to its rarity, subtle symptoms, and non‐specific imaging. Clinicians should maintain a high index of suspicion to facilitate early diagnosis and referral to dedicated sarcoma/breast units for optimal management of this rare malignancy.

## 1 Introduction

Angiosarcomas are rare and aggressive malignant endothelial cell tumours of vascular or lymphatic origin [1]. Breast angiosarcoma accounts for < 1% of all breast malignancies but represents one of the most common histological subtypes of breast sarcomas [2]. Breast angiosarcoma can be divided into primary (de novo) or secondary (therapy‐related) types [2]. Primary angiosarcoma (PAS) develops as a primary breast malignancy and is usually seen in younger females between 20 and 50 years old [2, 3]. Secondary angiosarcoma (SAS) occurs in patients who have had previous treatment for breast cancer, typically involving radiotherapy [1, 2].

Diagnosis of angiosarcoma is challenging due to a seemingly innocuous clinical presentation and variation in histopathological features [1, 3]. PAS typically presents as a breast mass without skin changes [2]. In contrast, SAS often appears as a bruise or purplish‐red papule, which can be mistaken for a benign lesion, frequently resulting in delayed diagnosis [1]. Imaging findings in both PAS and SAS are generally non‐specific, especially when using conventional breast screening methods such as mammography and ultrasound [4, 5]. While most patients initially present with localised disease, delays in diagnosis can lead to metastatic spread, which is associated with a poor prognosis [1]. Clinical vigilance and awareness of the disease are the key requirements for timely diagnosis of breast angiosarcoma. Due to the rarity of this malignancy, the number of published studies remains limited. This study aims to report a single institutional experience with both forms of breast angiosarcoma in a specialised breast/surgical oncology unit.

## 2 Methods

This was a retrospective, single‐centre study of patients with histologically proven angiosarcoma of the breast or (in the case of previous mastectomy) chest wall seen and surgically operated on at Mater Hospital Brisbane and Mater Private Hospital Brisbane between 2009 and 2024. PAS was defined as a malignant vascular neoplasm arising within the breast parenchyma, and SAS was defined as angiosarcoma arising within a previously irradiated field or in the context of a prior history of breast cancer, in accordance with the WHO definition [6].

Patients were identified through a prospectively maintained surgical logbook and medical records. Those without a pathology‐confirmed diagnosis of angiosarcoma were excluded, including patients with atypical vascular lesions or other primary breast sarcomas such as malignant phyllodes tumours with sarcomatous degeneration. Study data were collected from both public and private patient medical records. The study was approved by the Mater Misericordiae Ltd. Human Research Ethics Committee.

## 3 Statistical Analysis

Descriptive statistics were reported as means and percentages. Categorical variables were presented as counts and percentages, while continuous variables were reported as means and ranges. Associations between categorical variables were evaluated using Fisher's exact test or the chi‐square test, as appropriate. Independent samples *t*‐tests were used to compare continuous variables between groups. A *p* value of < 0.05 was considered statistically significant. All analyses were performed using Jamovi software [7].

## 4 Results

### 4.1 Patient Characteristics and Clinical Features

A total of 15 patients were included in the study, all of whom were female. Four patients had PAS and 11 had SAS. Patient characteristics and clinical features are shown in Table 1. Patients with PAS were younger than those with SAS (45.3 years vs. 69.1 years, *p* < 0.01).

**TABLE 1:** Patient characteristics and clinical features.

Description	PAS (*n* = 4)	SAS (*n* = 11)	*p*
Age, mean (range)	45.3 (35–63)	69.1 (53–82)	0.0014
Female, *n* (%)	4 (100)	11 (100)	1
Previous history of breast cancer, *n*			
Yes, *n* (%)	0 (0)	11 (100)	0.0001
No, *n* (%)	4 (100)	0 (0)	
Clinical presentation			
Skin bruising/discoloration/hematoma, *n* (%)	0 (0)	8 (72.7)	0.013
Palpable mass, *n* (%)	0 (0)	2 (18.2)	0.36
Nipple symptoms	2 (50)	0 (0)	< 0.01
Nipple discoloration, *n* (%)	1 (25)	0 (0)	
Recurrent mastitis/nipple infection, *n* (%)	1 (25)	0 (0)	
Incidental imaging finding, *n* (%)	1 (25)	1 (9.1)	0.42
Unknown	1 (25)	0 (0)	n/a
Average time of presentation to diagnosis, months (range)	1.1 (0.4–2)	15.2 (1–48)	0.13
Distant metastatic disease at time of diagnosis			
Yes, *n* (%)	0 (0)	2 (18.2)	0.36
No, *n* (%)	4 (100)	9 (81.8)	

**FIGURE 1:**
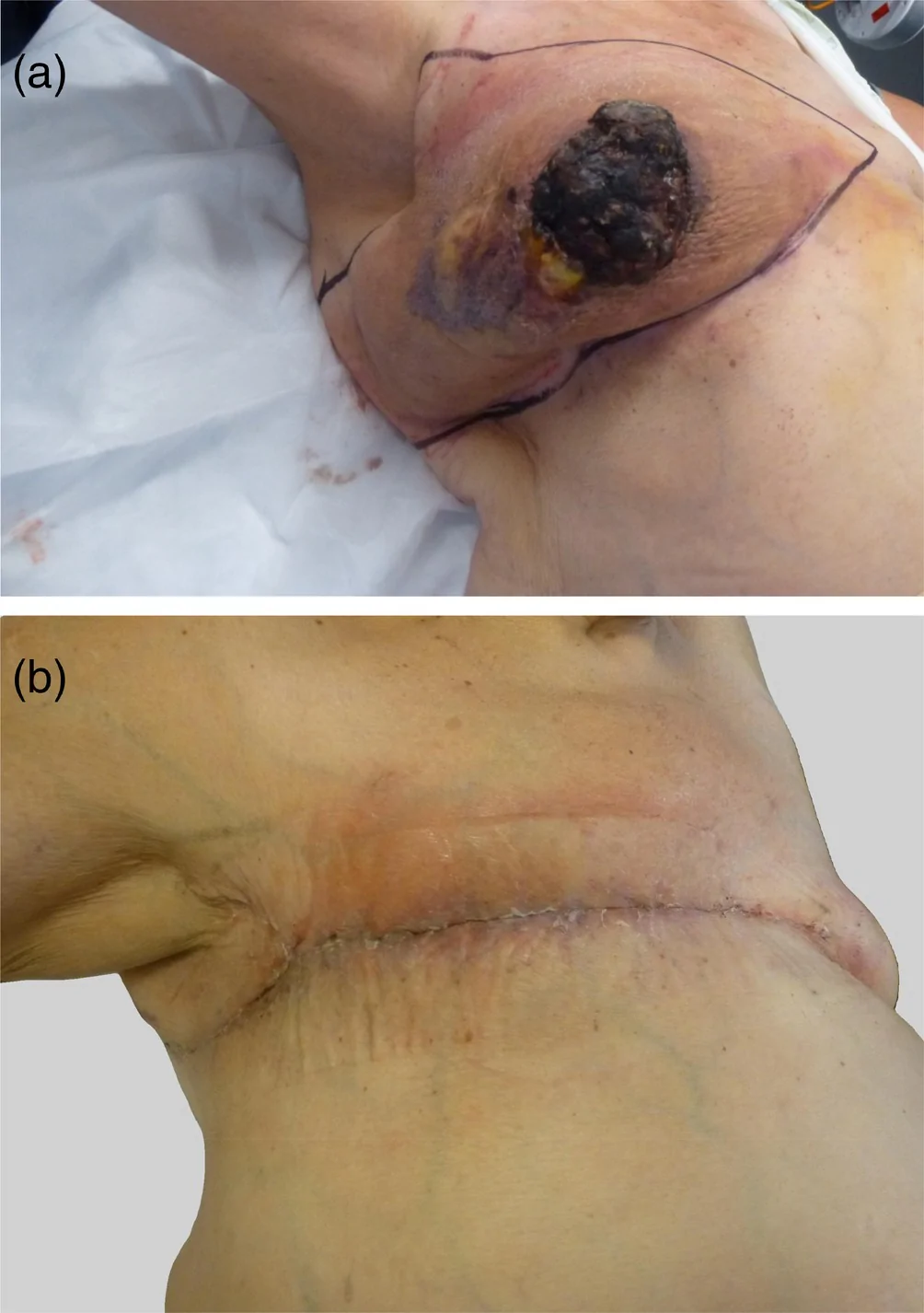
(a) Patient with primary angiosarcoma who presented with nipple discolouration, bruising and mass. She underwent wide local excision with primary closure. (b) Postoperative photograph of the patient 2 months after wide local excision and primary closure.

**FIGURE 2:**
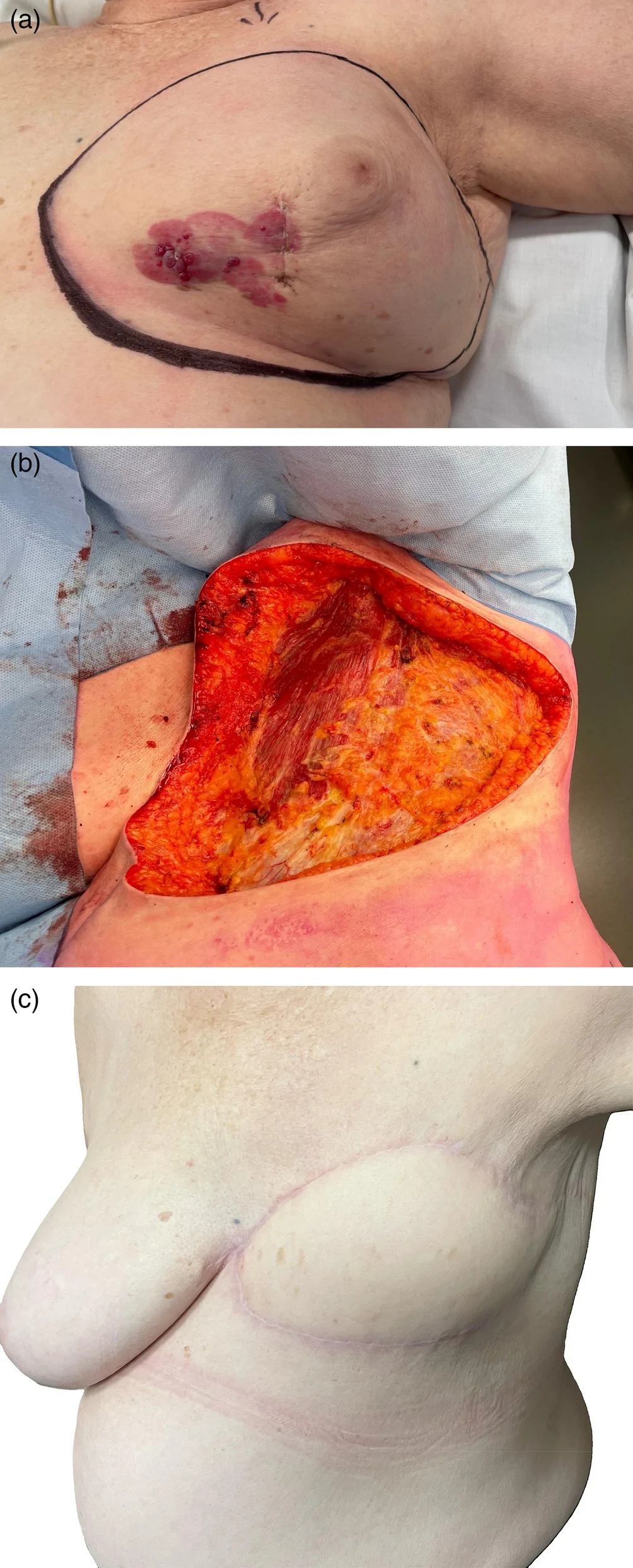
(a) Patient with characteristic features of secondary angiosarcoma—red skin discolouration with surrounding bruising, who underwent wide mastectomy with latissimus dorsi flap reconstruction. (b) Intraoperative photograph of the wide mastectomy performed to achieve clear margins. (c) Postoperative photograph of the patient 6 months after latissimus dorsi flap reconstruction.

The PAS group was more likely to present with nipple symptoms compared to breast symptoms in the SAS group (PAS = 50% vs. SAS = 0%, *p* < 0.01). One patient presented with nipple discolouration and one presented with nipple infection (Figure 1). The most common presenting symptom in the SAS group was skin discolouration (Figure 2), bruising, or haematoma (PAS = 0% vs. SAS = 72.7%, *p* = 0.013).

PAS patients were diagnosed earlier, with a median time to diagnosis of 1.1 months compared to 15.2 months for SAS patients; however, this difference was not statistically significant, likely due to the small sample size (*p* = 0.13). Additionally, 18.2% of patients in the SAS group had metastatic disease at presentation, compared to none in the PAS group (*p* = 0.36).

### 4.2 Breast Cancer History in Secondary Angiosarcoma

All patients in the SAS group had a previous history of breast cancer, as shown in Table 2. The mean tumour size was 21.2 mm, with an equal distribution of tumour grade and nodal status. The majority of patients had previously undergone lumpectomy and sentinel lymph node biopsy. All patients received adjuvant radiotherapy, with a mean interval of 10.1 years between treatment and diagnosis. Unfortunately, radiation dosage and field data were unavailable for analysis as the majority had treatment elsewhere. Most patients also received adjuvant chemotherapy (72.7%) and endocrine therapy (90.9%) as part of their primary breast cancer treatment.

**TABLE 2:** Previous ipsilateral breast cancer history in SAS.

Description	*N* = 11
Maximum tumour size (mm), mean (range)	21.2 (7–60)
Grade	
1	2 (18.1)
2	3 (27.3)
3	3 (27.3)
Unknown	3 (27.3)
Nodal status	
Positive	4 (36.4)
Negative	3 (27.3)
Unknown	4 (36.4)
Type of breast surgery	
Lumpectomy	9 (81.8)
Mastectomy	2 (18.2)
Type of axillary surgery	
Sentinel lymph node biopsy	6 (54.5)
Axillary lymph node dissection	5 (45.5)
Adjuvant radiotherapy	
Yes, *n* (%)	11 (100)
No, *n* (%)	0 (0)
Average time from radiation to diagnosis, years (range)	10.1 (3–26)
Adjuvant chemotherapy	
Yes, *n* (%)	8 (72.7)
No, *n* (%)	3 (27.3)
Unknown, *n* (%)	0 (0)
Adjuvant endocrine therapy	
Yes, *n* (%)	10 (90.9)
No, *n* (%)	0 (0)
Unknown, *n* (%)	1 (9.1)

### 4.3 Investigation

There was no significant difference in the number of investigations performed between the two groups. The mean number of investigations was 2 (range = 2) in the PAS group compared to 2.3 (range = 1–8) in the SAS group (*p* = 0.85). The PAS group had an average of one biopsy (range = 1), while the SAS group had an average of 2.1 biopsies (range = 1–5) (*p* = 0.21). Immunohistochemistry staining such as MYC, ERG, and CD31 were positive in most of the cases. All patients underwent staging PET/CT scans as part of their workup. Further details of investigations performed in the SAS group are shown in Table 3. Some patients had multiple prior investigations before referral to our centre, but these were not recorded.

**TABLE 3:** Further details of investigations in SAS group.

Patient	Number of imaging investigations	Modalities	Number of biopsies	Positive
1	8	US—Normal US—Normal US/MMG—Normal US—3 mm vascular lesion MMG—Resolving haematoma Contrast enhanced MMG—Normal MMG/US: Large well circumscribed lesion CT: Large 6 cm mass	3	Core biopsy 1—Negative FNA—Negative Core biopsy 2—AS
2	1^a^	MMG/US—Normal	5	Punch biopsy—Normal FNA—Normal Core biopsy 1—Normal Core biopsy 2—AS
3	2^a^	MRI—Large suspicious mass CT—Large mass	4	Core biopsy 1—Normal Core biopsy 2—Normal Core biopsy 3—Extensive necrosis Incisional biopsy—AS
4	2	US—Normal US and MRI—Suspicious mass	1	Incisional biopsy—AS
5	2	MMG/US – Fat necrosis right chest wall MRI – Oedema of skin and enhancement? Post surgical change	2	Punch biopsy—Normal Excisional biopsy—AS
6	2	US—Focal skin thickening MMG/US—Skin thickening	1	Excisional biopsy—AS
7	1	US—Skin thickening and collection suggestive of abscess	1	Incisional biopsy—AS
8	2	US/MMG—Dermal thickening US/MMG—Skin retraction, lymphedema and suspicious lesion	2	FNA—Benign Core biopsy—AS
9	1	US/MMG—Suspicious mass	2	Core biopsy—Atypical Excisional biopsy—AS
10	2	US—Normal MMG/US—Skin thickening and suspicious nodule	1	Punch biopsy—Suspicious
11	2	MMG/US—Normal MRI—Suspicious mass	1	Core biopsy—AS

^a^ Had numerous prior imaging from time of presentation but results were not documented in chart.

### 4.4 Management

**FIGURE 3:**
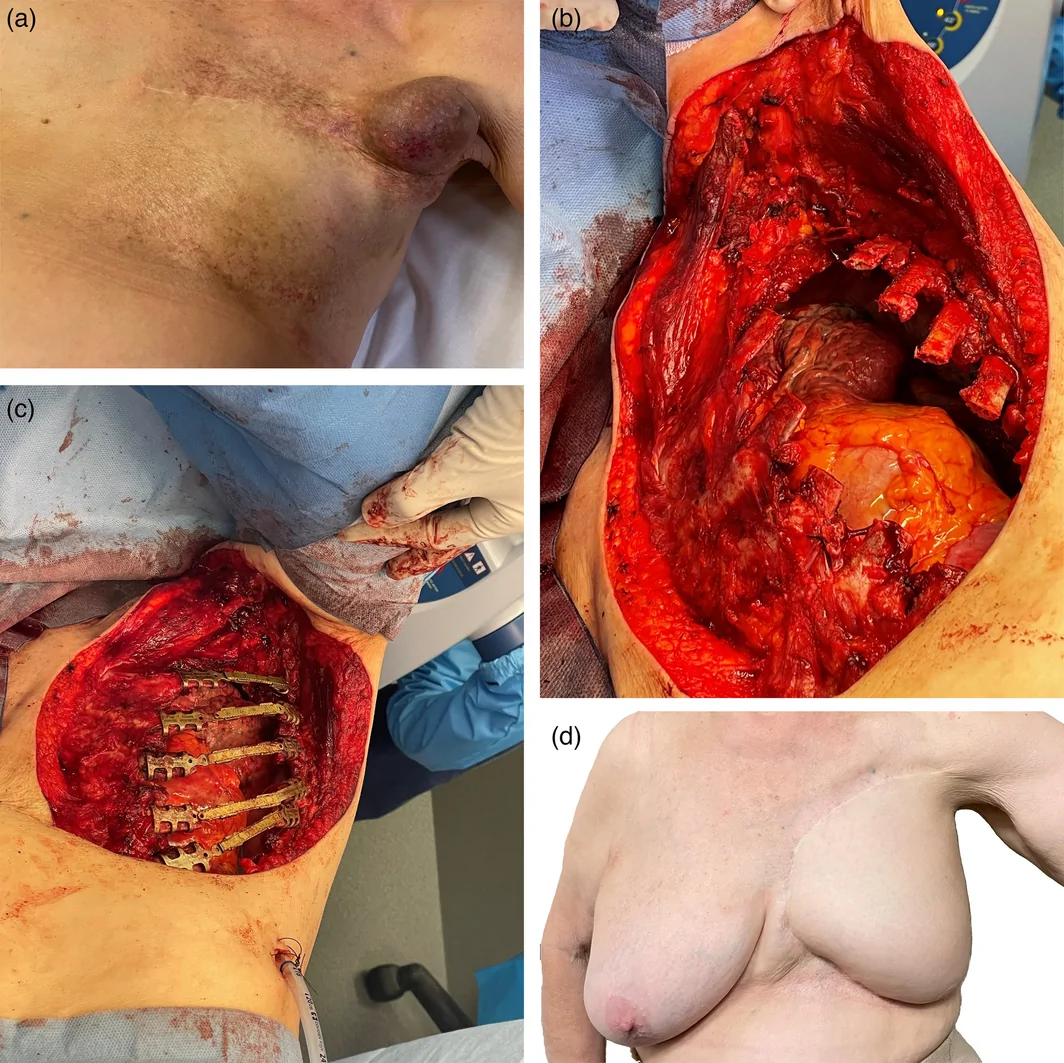
(a) Clinical photograph of a patient with secondary angiosarcoma presented with slowly progressive and infiltrative mass at the lateral aspect of her previous mastectomy scar. (b) Intraoperative photograph of the patient undergoing a wide mastectomy and rib resection to obtain clear margins. (c) Intraoperative photograph of the patient after undergoing rib reconstruction. (d) Clinical photograph of the patient 23 months after wide mastectomy, rib resection and reconstruction and free flap reconstruction.

All patients in the SAS group underwent mastectomy, compared to only 50% in the PAS group (*p* = 0.012). Of the 11 patients who had mastectomy in the SAS group, 5 underwent reconstruction (Figures 2b,c and 3a–d). Both patients in the PAS group who had mastectomy also had reconstruction at the same time. There were no postoperative complications in the PAS group. However, three patients in the SAS group experienced postoperative complications requiring revisional surgery (wound dehiscence = 2, empyema = 1).

There were no significant differences in maximum tumour size between the two groups (PAS = 52.8 mm vs. SAS = 71 mm, *p* = 0.54) or in immunohistochemistry staining. The majority of patients did not receive adjuvant treatment. One patient in the SAS group was lost to follow‐up, and there was no record of adjuvant treatment for this case. Management of angiosarcoma in both groups is shown in Table 4.

**TABLE 4:** Management.

Description	PAS (*n* = 4)	SAS (*n* = 11)	*p*
Surgery			
Lumpectomy, *n* (%)	2 (50)	0 (0)	0.012
Mastectomy without reconstruction, *n* (%)	0 (0)	6 (54.5)	
Mastectomy with reconstruction, *n* (%)	2 (50)	5 (45.5)	
Local flap rotation	1	3	
Free flap	0	2	
Implant reconstruction	1	0	
Postoperative complications, *n* (%)			
Total	0 (0)	3 (27.3)	0.24
Wound dehiscence requiring revision surgery	n/a	2	
Empyema requiring decortication	n/a	1	
Maximum tumour size (mm), mean (range)	52.8 (11–160)	71.0 (5–150)	0.54
IHC			
MYC positive, *n* (%)	1 (25)	6 (54.5)	0.57
ERG positive, *n* (%)	1 (25)	8 (72.7)	0.24
CD31 positive, *n* (%)	1 (25)	6 (54.5)	0.57
Adjuvant radiotherapy			
Yes, *n* (%)	2 (50)	3 (27.3)	0.48
No, *n* (%)	2 (50)	7 (63.6)	
Unknown, *n* (%)	0 (0)	1 (9.1)	
Adjuvant chemotherapy			
Yes, *n* (%)	1 (25)	4 (36.4)	0.60
No, *n* (%)	3 (75)	6 (54.5)	
Unknown, *n* (%)	0 (0)	1 (9.1)	

### 4.5 Angiosarcoma Recurrence and Survival Outcomes

The median follow‐up duration was 27 months in the PAS group compared to 12 months in the SAS group, as shown in Table 5. In the PAS group, one patient developed distant metastatic disease at the 6‐month follow‐up and subsequently passed away. There was no local recurrence in the PAS group.

**TABLE 5:** AS recurrence and survival outcomes.

Description	PAS (*n* = 4)	SAS (*n* = 11)	*p*
Duration of follow up (months), median (range)	32.7 (2–48)	19 (1–48)	0.30
Outcome			
No evidence of recurrence, *n* (%)	3 (75)	7 (63.6)	0.68
Recurrence, *n* (%)	1 (25)	4 (36.4)	
Local recurrence, *n* (%)	0 (0)	2 (18.2)	0.36
Distant metastatic disease, *n* (%)	1 (25)	3 (27.3)	0.83
Died due to disease, *n* (%)	1 (25)	2 (18.2)	0.77

In the SAS group, four patients developed recurrence of disease (local and distant metastatic disease). One patient developed local recurrence in the chest wall at the 4‐month follow‐up and underwent further surgical resection with no further evidence of recurrence of disease at the time of this review (6‐month follow up). Another patient developed both local recurrence and distant metastatic disease (lung metastases) at the 8‐month follow‐up and subsequently passed away. Two patients developed distant metastatic disease at 36 months (stable lung metastases, alive) and 12 months (lung and bone metastases, deceased), respectively.

Therefore, the overall disease‐free survival rate was 75% in the PAS group and 63.6% in the SAS group (*p* = 0.68). The overall survival rate was 75% in both the PAS and SAS groups (*p* = 0.77). The Kaplan–Meier survival plot is shown in Figure S1. The median survival time could not be calculated due to the short follow‐up period.

## 5 Discussion

Breast angiosarcoma remains a diagnostic challenge due to its rarity, subtle clinical presentation, and heterogeneous imaging findings. The annual incidence of breast angiosarcoma is low, at 4.6 cases per million, but has been steadily increasing by about 4% per year [8]. Overall, the increase in SAS is higher than that of PAS, as observed in studies based on the SEER database [8, 9, 10]. Over a 15‐year period in our breast/sarcoma unit, we observed a similar trend, with the incidence of SAS being three times higher than that of PAS.

Due to the increasing use of breast‐conserving surgery and radiation therapy in the treatment of breast cancer [8, 9], we hypothesize that this has led to an increased incidence of SAS. Our study is consistent with previous research identifying lumpectomy as an independent risk factor for the development of angiosarcoma [8, 9], with 80% of patients in the SAS cohort having a history of lumpectomy. However, this association is likely confounded by the fact that all lumpectomy patients also received adjuvant radiotherapy. Whilst radiation dosage and field also appear to influence the development of angiosarcoma [11], unfortunately we were unable to analyse this in our study due to lack of available data. Additionally, altered lymphatic drainage following breast surgery may lead to breast lymphedema, which increases the risk of developing angiosarcoma, similar to lymphangiosarcoma described in Stewart‐Treves syndrome. Although both radiation therapy and breast lymphedema are independent risk factors, there may be a synergistic effect between the presence of lymphedema in an irradiated field that predisposes patients to the development of SAS [1, 2] with lymphedema acting as the primer and radiotherapy as the accelerant. With the emergence of radiotherapy de‐escalation trials such as EXPERT [12] and PROSPECTIVE trial [13], it will be interesting to observe whether the incidence of SAS declines over time. Additionally, certain familial breast cancer syndromes involving mutations in DNA repair genes, such as BRCA1 and BRCA2, have also been reported as predisposing factors for developing SAS after breast cancer treatment [1].

Our study supports the evidence that, while both PAS and SAS are types of breast sarcomas, they represent two distinct disease entities with differing clinical characteristics. PAS tends to occur in younger patients, with a mean age of 45.3 years in our study, consistent with previously published data [2, 8]. PAS usually presents as a breast mass without skin changes, whereas SAS typically presents with cutaneous signs such as bruising, ecchymoses, or a violaceous skin rash in the setting of prior radiotherapy for breast cancer and no underlying parenchymal mass [2, 14]. In our study, most SAS patients exhibited characteristic skin changes, while PAS patients predominantly presented with nipple‐related symptoms. Due to the small sample size, drawing definitive conclusions about these differing clinical features is challenging. Nevertheless, our findings highlight the diagnostic difficulty of angiosarcoma based solely on clinical presentation, given its heterogeneous manifestations and resemblance to benign breast conditions.

Traditionally, breast angiosarcoma is challenging to diagnose with breast imaging due to nonspecific radiologic features. Mammographic findings can include a mass without calcification, breast asymmetry, skin thickening, or the lesion may be mammographically occult [4, 15]. Breast MRI has improved the sensitivity for detecting breast PAS, often revealing a characteristic rapid enhancement with washout post‐contrast imaging and diffuse high‐intensity skin thickening on T2‐weighted images [4, 15]. Our study showed that all patients who underwent breast MRI had suspicious findings, while some patients who had conventional breast imaging (mammography and ultrasound) had findings suggestive of a benign aetiology, thus delaying diagnosis of angiosarcoma. It is likely that some patients had prior breast imaging at symptom onset before referral to our centre, but this information was unrecorded, preventing meaningful retrospective analysis.

Due to radiological challenges, obtaining accurate histopathological tissue is essential for diagnosing angiosarcoma of the breast. However, core biopsy, often performed as a first‐line investigation for breast lesions, is associated with significant underestimation of breast angiosarcoma, likely due to insufficient tissue, fragmented specimens, and overlapping features between benign and well‐differentiated malignant vascular lesions [16]. SAS is also typically a cutaneous phenomenon; thus, both skin and lesion biopsies are needed. Most patients in our study required multiple core biopsies followed by incisional biopsy to obtain a definitive diagnosis, lengthening time to diagnosis. Immunohistochemistry and genetic molecular analysis can aid in histopathological diagnosis. SAS is often positive for amplification of c‐MYC, a proto‐oncogene, while PAS harbors mutations in PIK3CA and KDR. Overexpression of CD117 and CD31, markers of neoplastic endothelial cells, is often positive in both types of angiosarcoma [17, 18].

Breast angiosarcoma is an aggressive malignancy with a poor prognosis. The overall 5‐year survival rate ranges from 47% to 49% in PAS and 42% to 45% in SAS [3, 8]. Although our overall survival rates are higher (75% in the PAS group and 81.8% in the SAS group), our median follow‐up periods are shorter, at 32.7 months for PAS and 19 months for SAS. We expect the overall survival rates to decline over time. Nevertheless, during our relatively short follow‐up, we have shown that angiosarcoma remains an aggressive disease, with low disease‐free survival rates of 75% in the PAS group and 63.6% in the SAS group. Poor prognostic factors include large tumour size and high grade [2, 14]. While some studies suggested increasing age is associated with poorer outcomes [2, 9], others have found no significant correlation [14].

The mainstay of treatment for angiosarcoma remains surgical resection with clear margins. It is still debated whether more aggressive surgery with mastectomy is always necessary, or if breast‐conserving surgery combined with adjuvant treatment offers comparable survival outcomes. Studies have shown no survival advantage with mastectomy [2, 14]. In PAS, achieving clear margins with breast‐conserving surgery is feasible if the diagnosis is made early enough to allow for lumpectomy. However, in SAS, the extent of skin involvement typically encompasses the entire radiotherapy field, making breast‐conserving surgery unfeasible. Wide mastectomy with reconstruction is usually required to achieve oncological margins while maintaining acceptable cosmetic outcomes as can be seen in Figure 3a–d. In our study, mastectomy was the preferred surgical option for SAS, while lumpectomy was performed in 50% of PAS patients.

Other treatment modalities, such as chemotherapy and radiotherapy, are controversial and should be considered on a case‐by‐case basis within a dedicated sarcoma multidisciplinary team (MDT). Adjuvant chemotherapy and radiotherapy are associated with improved recurrence‐free survival, especially in higher‐grade disease [2, 14]. Re‐irradiation should be considered for selected SAS patients due to the high risk of local recurrence [2, 9], although this must be balanced against increased treatment‐related morbidity, such as pneumonitis and rib fractures, resulting from higher cumulative radiation doses. Both chemotherapy and radiotherapy have been used in the neoadjuvant setting, though their benefits remain unclear [14, 19]. Management of angiosarcoma within a dedicated sarcoma unit and discussion of cases in sarcoma MDTs have been shown to improve prognosis [3].

Our study has several limitations. The data were analysed retrospectively, making the study susceptible to reporting bias. Due to the rarity of the disease and the single‐centre nature of our sarcoma unit, the sample size is small. Consequently, we were unable to perform meaningful statistical analyses, such as univariate and multivariate analyses, to investigate risk factors for developing angiosarcoma or clinicopathological features that may influence prognosis. Therefore, we cannot provide treatment recommendations based on our findings.

Nonetheless, our study contributes valuable information on this rare tumour and supports previously published data indicating that angiosarcoma is an aggressive malignancy. Diagnosis is often delayed due to subtle clinical presentations being missed, occult findings on breast imaging, and difficulty in obtaining a tissue diagnosis. In patients with a previous history of breast cancer or chest wall radiation, we recommend that physicians maintain a high index of clinical suspicion when patients present with skin or nipple changes as described in our study. Dedicated breast imaging, including breast MRI, should be promptly organised, along with tissue diagnosis in the form of a core or incisional biopsy. Prompt diagnosis facilitates referral to a dedicated sarcoma or surgical oncology unit for appropriate management. PAS may be managed with breast conservation, while SAS typically requires wide mastectomy and flap reconstruction.

## 6 Conclusion

Breast angiosarcoma is a rare but aggressive malignancy with a high rate of local and systemic recurrence. Diagnosis is challenging due to non‐specific imaging findings and high false‐negative rates with core biopsies. The characteristic clinical presentation of skin changes (bruising or violaceous rash) in patients with a personal history of breast cancer should alert clinicians to the possibility of SAS. Surgical resection with clear margins remains the gold standard treatment for breast angiosarcoma.

## Author Contributions

**Gaik Si Quah:** conceptualization, investigation, writing – original draft, methodology, visualization, validation, writing – review and editing, software, formal analysis, project administration, data curation, resources. **Christopher Pyke:** writing – review and editing, conceptualization, supervision, data curation.

## Supporting information


**Figure S1:** The Kaplan Meier (survival) plot of time (in months) after angiosarcoma diagnosis, categorised by subtype. There was insufficient data to calculate median survival time.

## Data Availability

Research data are not shared.
