# Supplementary material for: Breast Angiosarcoma: 15‐Years Experience
Source: ANZ J Surg. 2026 Apr 16;96(7-8):2063–71. doi: 10.1111/ans.70673 (PMC13536265; doi:10.1111/ans.70673)
Supplement: Supplementary file 1 [file ANS-96-2063-s001.docx]

**Supplementary Figures**

**Supplementary Figure 1: The Kaplan Meier (survival) plot of time (in months) after angiosarcoma diagnosis, categorised by subtype. There was insufficient data to calculate median survival time.**

## Survival Curve
